# 642. mRNA-1608, an mRNA-Based Therapeutic Genital Herpes Vaccine Candidate: Interim Safety, Immunogenicity and Clinical Endpoint Results from a Phase 1/2, Randomized, Observer-Blind, Controlled, Dose-Ranging Trial

**DOI:** 10.1093/ofid/ofaf695.206

**Published:** 2026-01-11

**Authors:** Anna Wald, Christy A Comeaux, Alan Embry, Kenneth H Mayer, Rekha R Rapaka, Lei Gao, Zhantao Lin, Philip Buck, Sumana Chandramouli, Jamie Rosen, Jessica Winer, Adrienne Dunn, Haixing Wang, Alec Freyn, Robert Paris

**Affiliations:** University of Washington, Seattle, WA; Moderna Inc, Cambridge, Massachusetts; Moderna Therapeutics, Cambridge, Massachusetts; Fenway Health, Boston, MA; Moderna Therapeutics, Cambridge, Massachusetts; Moderna Inc, Cambridge, Massachusetts; Moderna Inc, Cambridge, Massachusetts; Moderna, Inc., Cambridge, Massachusetts; Moderna, Inc., Cambridge, Massachusetts; Moderna Inc, Cambridge, Massachusetts; Moderna Inc, Cambridge, Massachusetts; Moderna Inc, Cambridge, Massachusetts; Moderna Inc, Cambridge, Massachusetts; Moderna Inc, Cambridge, Massachusetts; Moderna, Inc., Cambridge, Massachusetts

## Abstract

**Background:**

HSV-2 is a common infection prevalent in approximately 13% of the global population and the leading cause of recurrent genital ulcers. mRNA-1608 is being developed as a therapeutic vaccine aimed to reduce genital herpes disease in persons with recurrent HSV-2. mRNA-1608 is an LNP dispersion containing 5 different mRNAs encoding modified versions of 3 HSV-2 glycoprotein antigens (gB, gC, and gD) and 2 immediate early proteins (ICP0 and ICP4). These 5 proteins are known targets of immune responses in healthy individuals following natural infection. Study mRNA-1608-P101 was designed to generate safety and immunogenicity data and establish a proof-of-concept of clinical benefit of the mRNA-1608 vaccine candidate.

**Methods:**

mRNA-1608-P101 is a Phase 1/2, randomized, observer-blind, controlled, dose ranging-study of mRNA-1608 in approximately 300 healthy adults 18 to 55 years of age with recurrent genital HSV-2 infection. Participants were assigned in a 1:1:1:1 ratio to receive mRNA-1608 at 1 of 3 dose levels (25, 50 and 100 μg) or control meningococcal Group B vaccine administered as 2 doses at 0 and 2 months. The primary objective was to evaluate safety and reactogenicity of mRNA-1608. Additional endpoints included immunogenicity and select clinical endpoints. Interim analysis results are reported here through 6 months post-dose 2.

**Results:**

A total of 302 participants were randomized and received the first injection, and 292 participants received the second injection. No safety concerns were identified. The majority of solicited adverse reactions were grade 1 or 2 in severity. Antigen-specific binding antibody responses (gB, gC and gD) and nAbs against HSV-2 were increased for all mRNA-1608 study groups. mRNA-1608 also elicited cell-mediated immune responses, primarily CD4+ type, against all 5 antigens. In exploratory analyses, dosing with mRNA-1608 showed a trend for delay in time to first genital herpes recurrence and a decrease in recurrence rate relative to control vaccine.

**Conclusion:**

Interim data from this Phase 1/2 study show that mRNA-1608 was generally safe and well-tolerated and induced antigen-specific immune responses. Administration of mRNA-1608 showed a trend of improvement in select clinical endpoints of recurrent genital herpes disease.

**Disclosures:**

Kenneth H. Mayer, MD, Gilead Biosciences: Advisor/Consultant|Gilead Biosciences: Grant/Research Support|GSK/ViiV Healthcare: Advisor/Consultant|GSK/ViiV Healthcare: Grant/Research Support|Merck and Company, Inc.: Advisor/Consultant|Merck and Company, Inc.: Grant/Research Support|Moderna, Inc.: Grant/Research Support

